# Neural representation of calling songs and their behavioral relevance in the grasshopper auditory system

**DOI:** 10.3389/fnsys.2014.00183

**Published:** 2014-12-19

**Authors:** Gundula Meckenhäuser, Stefanie Krämer, Farzad Farkhooi, Bernhard Ronacher, Martin P. Nawrot

**Affiliations:** ^1^Neuroinformatics and Theoretical Neuroscience, Department of Biology, Chemistry and Pharmacy, Institute of Biology, Freie Universität BerlinBerlin, Germany; ^2^Behavioural Physiology Group, Department of Biology, Humboldt-Universität zu BerlinBerlin, Germany; ^3^Bernstein Center for Computational NeuroscienceBerlin, Germany

**Keywords:** acoustic communication, decision making, naïve Bayes classifier, neural information processing, pattern recognition, population coding

## Abstract

Acoustic communication plays a key role for mate attraction in grasshoppers. Males use songs to advertise themselves to females. Females evaluate the song pattern, a repetitive structure of sound syllables separated by short pauses, to recognize a conspecific male and as proxy to its fitness. In their natural habitat females often receive songs with degraded temporal structure. Perturbations may, for example, result from the overlap with other songs. We studied the response behavior of females to songs that show different signal degradations. A perturbation of an otherwise attractive song at later positions in the syllable diminished the behavioral response, whereas the same perturbation at the onset of a syllable did not affect song attractiveness. We applied naïve Bayes classifiers to the spike trains of identified neurons in the auditory pathway to explore how sensory evidence about the acoustic stimulus and its attractiveness is represented in the neuronal responses. We find that populations of three or more neurons were sufficient to reliably decode the acoustic stimulus and to predict its behavioral relevance from the single-trial integrated firing rate. A simple model of decision making simulates the female response behavior. It computes for each syllable the likelihood for the presence of an attractive song pattern as evidenced by the population firing rate. Integration across syllables allows the likelihood to reach a decision threshold and to elicit the behavioral response. The close match between model performance and animal behavior shows that a spike rate code is sufficient to enable song pattern recognition.

## Introduction

**Figure 1:**
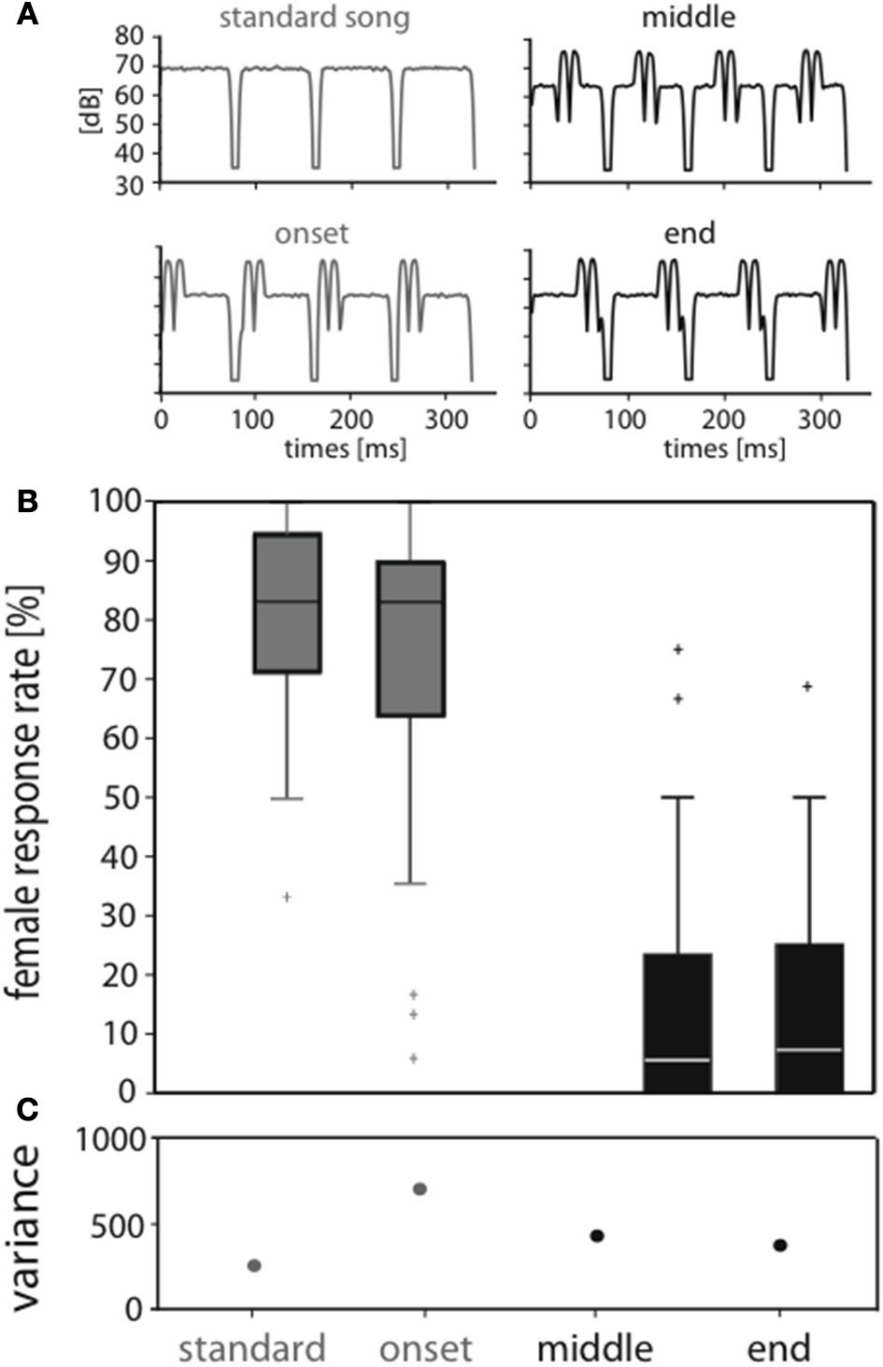
**Perturbation of the standard song affects attractiveness when placed at later syllable positions. (A)** Envelopes of song models used for behavioral and neurophysiological tests. An attractive standard song consisted of 72 ms syllables and 12 ms pauses. The other stimuli had the same syllable and pause durations but exhibited perturbations at different positions within a syllable (onset, middle, end). **(B)** The median response rate of 33 *C. biguttulus* female responses for the stimulus with onset perturbation was 83%, thus very similar to the response to the standard stimulus. In contrast, stimuli with perturbation in the middle and end were mostly rejected (median response rate 6%). The median is displayed as the central mark in the box plot. The edges of the box are the 25 and 75th percentiles. **(C)** Note the high variance in female responses, especially when perturbation is at syllable onset.

Acoustic communication of grasshoppers has become a prominent model system to investigate principles of neuronal processing of acoustic stimuli. It provides the opportunity to study perceptual decision making in a comparatively simple nervous system. Grasshoppers produce acoustic signals, termed “songs,” to attract a mating partner. Natural songs consist of a repetition of stereotyped subunits with species-specific amplitude modulations of a broad carrier frequency band that are produced by moving the hind legs against the forewings (Von Helversen and von Helversen, 1997). Due to characteristic differences between grasshopper species the songs constitute an important barrier against hybridization. Both the song production and the song recognition are innate behaviors, and therefore we can be confident that the corresponding neuronal circuits are “hard-wired.” In behavioral tests one can use artificial song models that mimic and vary certain song features, and thereby explore which cues are crucial for song recognition (Von Helversen, 1972; Von Helversen and von Helversen, 1997, 1998). These experiments demonstrated that the decisive cues for song recognition reside in the temporal pattern of amplitude modulations, i.e., in a song's envelope. In the grasshopper *Chorthippus biguttulus*, the subject of this investigation, a very simple but highly attractive song model consists of a series of sound “syllables” separated by pauses (see Figure 1A). Using song models we can reduce the signal's complexity and compare the behavioral responses directly with the processing capacities of neurons at different stages of the auditory pathway.

The nervous system of grasshoppers offers an important advantage: it contains identifiable neurons that can be discriminated on the basis of their characteristic morphology (Römer and Marquart, 1984; Stumpner and Ronacher, 1991). Thus, specific processing properties can be assigned to groups of identified neurons in the auditory pathway. The first stage of auditory processing comprises three neuron classes: auditory receptor neurons, local neurons (LNs) and ascending neurons (ANs). The ears of grasshoppers are located on the sides of the first abdominal segment. A total of approximately 60 receptor neurons transduce the vibrations of the tympanum into series of action potentials that travel via the axons into the metathoracic ganglion complex, which houses the first auditory processing stage. There, axons make contact to various types of LNs—about 10–15 different types of LNs have been identified so far. The LNs then contact a set of about 20 types of ANs, the axons of which ascend to the animal's head, and constitute the sole auditory input to higher processing circuits and decision centers located in the brain (Ronacher et al., 1986; Bauer and von Helversen, 1987). Since the population of ANs constitutes a bottleneck for the information that is available to the brain, they will be in the focus of the present study. Remarkably, the auditory pathway including the ANs is highly conserved between different grasshopper species (Ronacher and Stumpner, 1988; Neuhofer et al., 2008). Not only are the neurons' morphologies extremely similar in two not related species (*C. biguttulus* and the locust *Locusta migratoria*), but homologous neurons also exhibit the same physiological properties and processing capacities—for a detailed description of the response types see (Römer and Marquart, 1984; Stumpner and Ronacher, 1991; Stumpner et al., 1991; Wohlgemuth and Ronacher, 2007). Neuhofer et al. (2008) have shown that auditory neurons of the locust respond in the very same manner to a song signal of *C. biguttulus* as do the homologous neurons of *C. biguttulus*; the similarity of responses has been quantified by the van Rossum metric. Only at the next processing stages, located in the brain, we expect to find neuronal networks that respond selectively to the species-specific song patterns. Due to the high interspecific similarity of the local and ascending neurons we can compare neuronal properties of the locust's neurons with behavioral data obtained with *C. biguttulus*.

The decision centers located in the female brain must evaluate whether a heard song follows the con-specific pattern and whether it is attractive enough to trigger a response song as the appropriate behavior. This task appears simple under ideal conditions, since the song patterns of different species differ considerably (Stumpner and von Helversen, 1994; Gottsberger and Mayer, 2007). However, in nature there are many factors that may degrade the acoustic signal on its way from sender to receiver. This aggravates the classification problem. Here we introduced perturbations of the signal envelope that strongly influenced behavioral decisions. Applying perturbations to the pattern of an attractive song model affected the signal's attractiveness as measured by the female response rates differently, depending on the specific position of a perturbation within a song syllable (Figure 1A). Presenting the same stimuli while performing intracellular recordings from identified neurons allowed to investigate the neural representation of the stimulus identity and of its behavioral relevance.

Using naïve Bayes classifiers (for review see Pouget et al., 2000; Quiroga and Panzeri, 2009) we specifically asked to what degree the acoustic stimulus can be decoded and whether the behavioral stimulus category can be predicted from the single-trial responses of single neurons and neuron populations. We introduce an abstract model of decision-making for triggering a behavior based on the sensory information encoded in the AN population firing rate during a single trial. This model accounts for the observed behavioral scores to different stimulus types.

## Materials and methods

### Animals

The behavioral tests were performed with females of *C. biguttulus*. The animals were reared as the filial generation (F1) from eggs of individuals collected as adults near Göttingen, Germany. After adult molt females and males were held separately in plastic cages to ensure virginity. In this species the females respond to a male's song with a song of their own, thereby indicating their readiness to mate. This response song is an ideal criterion showing that a female has identified a song as belonging to a potential conspecific mating partner.

Electrophysiological experiments were performed on locusts, *L. migratoria*, that were bought from a commercial supplier (for details of the breeding and keeping procedures see Schmidt et al., 2008; Stange and Ronacher, 2012). We can homologize identified neurons between the two species on the basis of their characteristic morphology (Römer and Marquart, 1984; Stumpner and Ronacher, 1991). The homologous auditory neurons of the thoracic ganglia show quantitatively similar response patterns in both species (Neuhofer et al., 2008). In these experiments songs or song models of *C. biguttulus* were presented to both species, and neurons of the locust showed the same responses as neurons of *C. biguttulus* although these songs have, of course, no relevance for the locust (see also Ronacher and Stumpner, 1988; Sokoliuk et al., 1989). On the basis of this strong homology we can use recordings from *L. migratoria* neurons and compare their spike patterns with behavioral responses of *C. biguttulus*.

### Acoustic stimuli

A digitally generated song envelope consisting of rectangular syllables of 72 ms duration separated by 12 ms pauses served as an attractive standard stimulus (Figure 1A). In order to systematically screen the detrimental effect of degradation at different syllable positions, we inserted perturbations of 24 ms either in the first, or in the middle, or in the last part of each syllable (Figure 1A). A perturbation consisted of 2 alternating accents and gaps, each of 6 ms duration and 12 dB higher or lower sound pressure relative to the syllable plateau. Earlier experiments had revealed that gaps within a syllable do markedly reduce the stimulus attractiveness; accentuations that occur at the end of a syllable have similar detrimental effects (Von Helversen, 1972, 1979; Ronacher and Stumpner, 1988; Von Helversen and von Helversen, 1997; for reviews see Ronacher et al., 2004; Ronacher and Stange, 2013).

The envelopes of all song models were convolved with the same carrier frequency (a broad band noise spectrum of 5–40 kHz). Sound intensity was calibrated with a half inch microphone (type 4133; Brüel and Kjær, Nærum, Denmark) and a measuring amplifier (type 2209, Brüel and Kjær) at the position of the animal. All four test patterns were presented with the same effective intensity (RMS) of 70 dB SPL; therefore, the peak and plateau intensities differed between stimuli (syllable plateau 70 dB for the standard stimulus and 65 dB for perturbed stimuli, Figure 1A). Yet, these intensities fall into the intensity range well accepted by *C. biguttulus* females (Von Helversen and von Helversen, 1994, 1997). The songs presented in the behavioral and electrophysiology tests comprised the same envelope structure but differed in length: 2772 ms (33 subunits; behavior) and 756 ms (9 subunits for electrophysiology), respectively.

### Behavioral experiments

Virgin *C. biguttulus* females were tested in a sound proof chamber at a constant temperature of 30 ± 2°C. The experiments were automatically conducted by a custom made program (written by M. Hennig in Labview 7.1, National Instruments) presenting songs in a pseudo-randomized order while recording the females' responses (for details of the apparatus and testing procedures see Schmidt et al., 2008). Each song was iterated 18 times. As a measure of stimulus attractiveness we used the percentage of responses normalized to the 18 presentations for each female. Out of these individual responses median response rates were calculated. Additionally, a negative control was presented, comprising the same carrier frequency and length as the standard signal, but lacking any syllable pause structure. In applying this negative control stimulus those females indicating a not discriminative behavior for song patterns could be detected. We therefore excluded from further analysis 11 of 44 females as they responded more than twice to the negative control. Applied statistic software was GraphPad Instat Version 3.06.

### Electrophysiological experiments

Auditory interneurons were recorded intracellularly in the frontal auditory neuropil of the metathoracic ganglion in both sexes of *L. migratoria*. During the experiments the torso of the animal was filled with a locust Ringer solution (Pearson and Robertson, 1981), to prevent the ganglia from drying. The temperature was kept constant at 30 ± 2°C. For the recordings we used glass microelectrodes (borosilicate, OØ = 1 mm, IØ = 0.58 mm, GC100F-10; Harvard Apparatus, LTD, USA), with capacities varying between 20 and 100 MΩ. They were filled with a fluorescent dye, a 3–5 % solution of Lucifer yellow (Sigma–Aldrich, Taufkirchen, Germany) in 0.5 M LiCl. Neural responses were amplified (10-fold, BRAMP-01 R, npi, USA) and recorded by a data-acquisition board (PCI-MIO-16E-4, 16 bit, National Instruments, USA) with a sampling rate of 20 kHz. The dye was injected into the recorded cell by applying hyperpolarizing current of 0.5–1 nA. Subsequently the thoracic ganglia were incubated in a fixation solution (4% paraformaldehyde), dehydrated and cleared in methyl salicylate. This procedure allowed an identification of the stained cells under a fluorescent microscope according to their characteristic morphology (Römer and Marquart, 1984; Stumpner and Ronacher, 1991).

Experiments were performed in a Faraday cage lined with reflection absorbing prisms. One of two speakers (frequency response 2–40 kHz, D21, Dynaudio, Denmark), which were placed laterally, at a distance of 30 cm from the animal's tympanal organ, emitted the sound signal. The acoustic stimuli were attenuated (PA5, Tucker-Davis Technologies, USA) and amplified (Raveland-XA600, Conrad Electronics, Germany). They were stored digitally and delivered by custom-made software (LabVIEW, National Instruments) using a 100-kHz D/A-conversion (PCI-MIO-16E-1, National Instruments). For this study ANs were analyzed which represent the third processing stage in the metathoracic ganglion and transmit the auditory information to the grasshopper's brain. We recorded four different types of ANs (AN1, AN4, AN3, AN12) from 25 animals (details of the response properties of these neurons can be found in Ronacher and Stumpner, 1988; Stumpner and Ronacher, 1991; Wohlgemuth and Ronacher, 2007). The direction from which the sound stimuli were presented depended on the side where the neurons were more sensitive to. With the exception of AN1 the ANs AN4, AN3, and AN 12 do not exhibit strong direction sensitivity. The AN1 was mostly recorded from the contralateral side (respective to the soma), the other neurons from both sides. Each song was presented within a looped order: standard stimulus, onset-perturbation, perturbation in the middle, then perturbation in the end, and starting again with the standard stimulus. Stimulus iteration was 8 times, each iteration comprised the full stimulus length (9 subunits).

### Data analysis

#### Estimation of firing rates and trial-by-trial variability

We estimated time-resolved firing rate profiles from single spike trains by convolution with a Gaussian kernel with width б ranging from 1 to 30 ms and support [−4·б,4·б] (Nawrot et al., 1999). The kernel was normalized to unit area such that the time integral of the estimated rates equals the number of spikes.

To quantify the trial-by-trial variability of the single neuron spike count we employed the commonly used measure of the Fano factor (Nawrot et al., 2008; Nawrot, 2010), which computes the variance of the spike count across repeated trials divided by the trial-averaged spike count within in a fixed observation interval.

#### Naïve Bayes classification

Naïve Bayes classifiers are statistical classifiers that are based on Bayes' theorem together with naïve independence assumptions. We applied Bayesian classifiers to decode which stimulus class evoked a particular neural response. Naïve Bayes classifiers have frequently been used to quantify encoded information in neural spike trains (for reviews see Pouget et al., 2000; Quiroga and Panzeri, 2009), for instance in olfactory sensory neurons in Drosophila larvae (Hoare et al., 2011), in visual interneurons of the blowfly (Karmeier et al., 2005), or in motor cortical neurons of behaving monkeys (Rickert et al., 2009). Let *P*(s) denote the probability of presentation of stimulus class s and *P*(*x*_1_, …, *x_n_*|*s*) the conditional probability of observing spike train features *x*_1_, …, *x_n_* given s. The posterior probability that stimulus class s was presented given *x*_1_, …, *x_n_* is according to Bayes' theorem

$$\begin{array}{l} P(s|x_{1},\ldots ,x_{n})=\frac{P(x_{1},\ldots ,x_{n}|s)}{P(x_{1},\ldots ,x_{n})}P(s),\text{with}\\ \;P(x_{1},\ldots ,x_{n})=\sum_{s\in S}P(x_{1},\ldots ,x_{n}|s)P(s). \end{array}$$

The naïve independence assumption that each feature x_i_ is conditionally independent of feature *x*_*j*_ given s simplifies to

$$P(s|x_{1},\ldots ,x_{n})=\frac{\prod_{i=1}^{n}P(x_{i}|s)}{P(x_{1},\ldots ,x_{n})}P(s).$$

From this posterior probability distribution the stimulus class $\hat{\text{s}}$ that maximizes the probability that *x*_1_, …, *x_n_* was observed is chosen:

$$\hat{s}=argmax_{s\in S}\{P(s|x_{1},\ldots ,x_{n})\}.$$

Since *P*(*x*_1_, …, *x_n_*) is constant for any choice of the stimulus class s, the classification rule can be written as

$$\hat{s}=argmax_{s\in S}\left\{\prod_{i=1}^{n}P(x_{i}|s)P(s)\right\}.$$

***Different decoding approaches***. First, we decoded stimulus classes based on the spike count of single neurons which can be considered as a very simple descriptor of a neural spike response pattern. For each stimulus of 756 ms duration we counted the number of spikes for each of the eight trials, which is proportional to the time-averaged firing rate over the total stimulus length. In a leave-one-out cross-validation every count c was used once as validation data to decoded the stimulus class as:

$$\hat{s}=argmax_{s\in S}\{P(c|s)P(s)\},$$

while the remaining counts were used as training data to compute the probability density functions *P*(c|s) with kernel density estimation. The estimation was implemented with scipy.stats.gaussian_kde (Oliphant, 2007). As the procedure includes automatic bandwidth determination, the probability density functions were estimated with different bandwidths. To account for the non-negativity of the counts, we restricted the support to positive values and normalized the probability density function to unit area. For the very rare case that not more than two counts had different values we assumed a Poisson distribution with mean of the counts.

Second, for decoding from a pseudo-population of neurons, we used the counts *c*_1_, …, *c_n_* of n neurons of different type recorded in different females and calculated

$$\hat{s}=argmax_{s\in S}\left\{\prod_{i=1}^{n}P(c_{i}|s)P(s)\right\}$$

to decode which stimulus class triggered the counts *c*_1_, …, *c_n_*.

***Grouping of stimuli into classes***. We followed the decoding approaches to first decode the four stimuli. In this case the set S of stimulus class consists of the standard stimulus, onset perturbation, middle-perturbed song, and end-perturbed song, i.e., each song forming a single class. As all four songs were equally often presented we applied the classification rules with *P*(s)=1/4 for all *s* ∈ *S*. However, we may also define stimulus classes that consist of grouped stimuli. For example, decoding whether or not a song shows degradation yields two classes, one consisting of the standard stimulus, and the other one of the three perturbed songs. The prior of these two classes is:

$$P(s)=\{\begin{array}{c} 1/4,\;for\;s=standard\;stimulus\;\\ 3/4,\;for\;s=perturbed\;stimulus \end{array}$$

***Performance of the Classifier***. To validate the performance of the classifier we performed a leave-one-out cross validation in which each single trial response was used once for decoding based on the distribution of the remaining trials. The results were stored in a confusion matrix (Jurman et al., 2012) whose entry (i,j) represents the number of times that a presentation of stimulus class *i* was predicted to be stimulus class *j*. Based on the confusion matrix we quantified the decoding performance with the Matthews correlation coefficient (MCC) as it is defined in Jurman et al. (2012). The MCC assumes values between −1 and 1, where 0 indicates chance level classification and 1 perfect prediction. In case of binary classification (e.g., decoding the standard stimulus against the three perturbed stimuli) the formula reads

$$MCC=\frac{TP\cdot TN-FP\;\cdot FN}{\sqrt{\left(TP+FP)(TP+FN)(TN+FP)(TN+FN\right)}}$$

where TP, TN, FP, and FN denote true positives, true negatives, false positives, and false negatives, respectively. The MCC has two advantages over the more common measure of accuracy = (TP + TN)/(TP + TN + FP + FN), commonly referred to as “fraction correct.” First, the MCC can be applied in multiclass problems even if the classes are of different sizes (Gorodkin, 2004; Jurman et al., 2012) whereas the measure of accuracy is biased in the case of uneven sample sizes. In our case the sample size is uneven when we group stimuli into classes. Second, the chance level of the MCC is 0 independent of the number *m* of classes whereas the chance level of accuracy (1/*m*) depends on the class number. In our case the MCC thus allows for a direct comparison of decoding performance for stimulus classification (3 or 4 different stimuli) and prediction of the behavioral state (2 classes: attractive or unattractive).

To test whether a classifier decodes significantly better than chance we performed a leave-one-out cross-validation based on spike train features that were randomly reassigned to the stimuli, followed by a calculation of the MCC. We repeated this procedure 1000 times and calculated the *p*-value as the percentage of MCCs that are larger than or equal to the actual MCC. A significance level of 0.05 was chosen.

We implemented all data analysis algorithms in the Python programming language.

### Model of decision making

Following Gold and Shadlen (2007) we use the experimental realizations of the count pattern in *n* = 8 ANs to fit a simple probabilistic model for the female's decision to respond to a calling song. This model is based on the log likelihood ratio (LR) of the song attractiveness given the AN population spike count. We computed for each syllable *j* separately the log LR as

$$\log LR_{\pm }(j)=\log\frac{P(c_{1}(j),\ldots ,c_{8}(j)|h_{+})}{P(c_{1}(j),\ldots ,c_{8}(j)|h_{-})}$$

where the denominator accounts for the probability that the a given count vector *c*_1_(*j*), …, *c*_8_(*j*) (test trial) across 8 neurons stems from the hypothesis h_+_, which is represented by the probability distribution of counts estimated from the remaining trials given an attractive stimulus s_+_. We then defined the decision variable as:

$$DV(k)=\sum_{j=1}^{k}\log LR_{\pm }.$$

The decision variable is updated after each syllable *k* by taking the cumulative sum over the past log LR values up to the *k*th syllable. It represents a cumulative sum over the evidence for the presence of an attractive song. The larger DV the more likely is the presence of an attractive song over an unattractive song.

For any combination of *n* = 8 selected ANs, two of each type, we compute for each single song presentation (test trial) the LR and the DV based on the remaining trials (leave-one-out). We repeat this for all possible combinations of 8 neurons that comprise 2 neurons of each type of AN representing input from both ears. We next introduced a decision threshold θ on DV. For a single trial, i.e., a particular song presentation, a behavioral response is elicited if DV(k) > θ in any *k*. This approach allows us to simulate the female single trial response behavior based on the experimentally recorded AN population activity.

We compared the performance of the simulated animal decisions to the actual animal performance in the behavioral experiments. For a given value of θ the true positive (TP) rate is defined as the fraction of correct detections, i.e., threshold crossings in the presence of an attractive song over all presentations of an attractive song. The false positive (FP) rate quantifies the fraction of false alarms, i.e., the threshold crossings in the presence of an unattractive song over all presentations of an unattractive song. TP and FP rates depend on the choice of θ. We thus computed the receiver operating characteristic (ROC) that represents the TP rate as a function of the FP rate for varying θ (Wiley, 2006). We measure the area under the ROC to quantify the model performance independent of the behavioral threshold θ.

## Results

### Behavioral decisions reveal two behaviorally relevant stimulus classes

In behavioral tests we investigated how degradation at specific positions within the signal does affect signal recognition. We compared the responses of *C. biguttulus* females to four stimulus types (Figure 1A): (i) standard stimulus without perturbation, (ii) with perturbation during the first third of the syllable (“onset”), (iii) during the second third (“middle”), and (iv) during the last third (“end”). Figure 1B shows the distribution of response rates across individual females to all four stimuli (see Materials and Methods). The standard stimulus was highly attractive (median: 83%), although individual females differed considerably in their response rate (compare quartile ranges and see variance in Figure 1C). Females showed similar high response rates toward the stimulus with onset perturbation, whereas the same perturbation in the middle or the end of a syllable led to a behavioral rejection (median response levels of <10%). Only 3 out of 33 females responded to the latter stimuli in more than 50% of the stimulus presentations.

**Figure 2:**
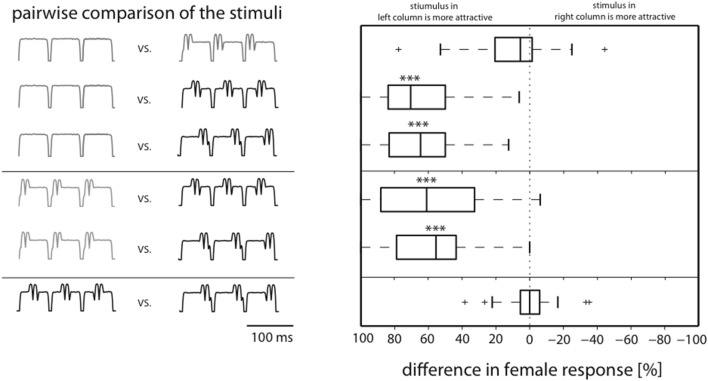
**Pairwise comparison of individual female responses allows distinction in attractive and unattractive stimulus classes**. Box plots show medians of response differences in individual females for stimulus comparisons shown in the left. Whereas there is no difference in response between stimuli with onset perturbation and the standard song, they are both significantly more attractive than stimuli with perturbation at middle and end (***p < 0.001, Dunn's *post-hoc* test after Friedman).

In order to further analyze differences in attractiveness we pairwise compared stimulus responses in individual females. For each female, the response rates for any two stimuli (see left column in Figure 2) were subtracted. Thus, it could be shown that the responses to the onset stimulus did not differ significantly from the responses to the standard (top row, Figure 2); the same is true for the comparison of the stimuli perturbed in the second and third part of the syllable (lowest row, Figure 2). In contrast, the responses to the unperturbed song and the song with middle and end perturbations differed significantly (*p* < 0.001; Friedman and Dunn's Multiple Comparison Test), and in both cases the median difference was about 60%. Similar results were found for the comparison between the onset perturbed stimulus and the other two perturbed stimuli (median differences >50%, *p* < 0.001).

### Decoding stimulus identity and behavioral class from the neuronal spike count

Grasshoppers have to make their decisions based on the information about the environment provided by the sensory and higher order neurons of the auditory pathway. The clear separation into two behavioral stimulus classes raises the question of how the different stimuli and the different behavioral classes are represented and discriminated within the grasshopper's nervous system. We address this question in intracellular *in vivo* recordings of identified ANs during repeated presentations of all four songs. To quantify the encoded information we apply a single-trial decoding approach to the neural spiking activity using a Bayesian classifier. We first decode the identity of the auditory stimulus before we predict the behavioral class (attractive vs. non-attractive).

#### Stimulus classification based on single neuron and population activity

**Figure 3:**
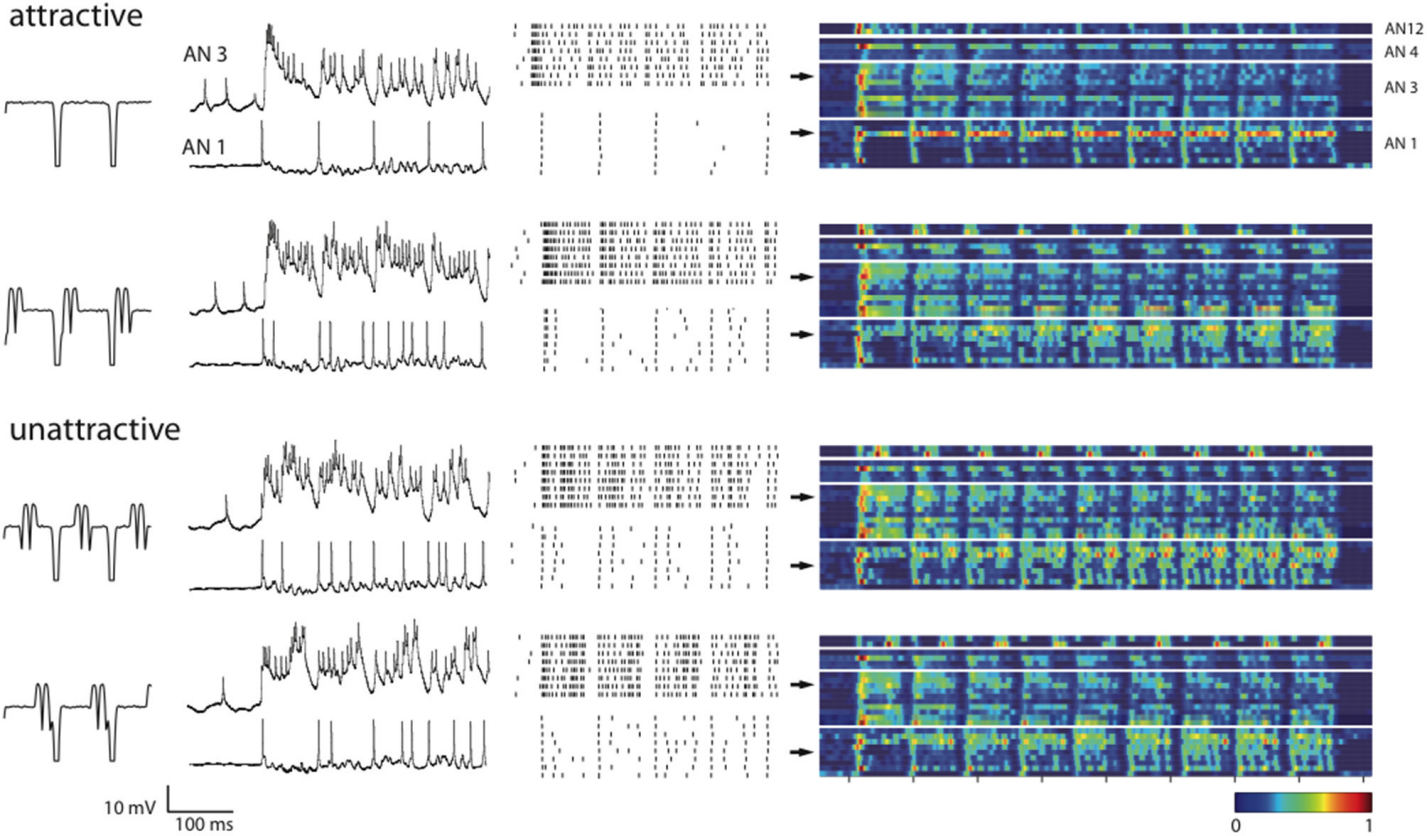
**Neuronal responses to all four songs categorized by their behavioral relevance**. Voltage traces and spike raster plots (8 trials) in the second and third columns show responses to the first four syllable–pause subunits for two example neurons AN3 and AN1. The fourth column shows trial-averaged firing rates estimated with a Gaussian kernel of width б = 4 ms during the whole stimulus presentation. Each row within a block of a neuron type represents the response of a single neuron [from top to bottom AN12 (*n* = 2), AN4 (*n* = 4), AN3 (*n* = 10), AN1 (*n* = 9)]. Color denotes the amplitude of the estimated firing rates normalized to the maximum rate within each neuron class. Arrows point out the firing rates of the shown examples.

How is information about a stimulus, such as the stimulus type or its attractiveness, represented in the spike responses of the ANs? We obtained intracellular recordings from AN1 (*n* = 9), AN3 (*n* = 10), AN4 (*n* = 4), and AN12 (*n* = 2); for the terminology see Römer and Marquart, 1984; Stumpner and Ronacher, 1991). Figure 3 shows example voltage traces of *in vivo* intracellular recordings from two individual ANs, and the corresponding spike raster plots. The example AN3-neuron responded with a burst of spikes to the stimulus onset and with smaller bursts at syllable onsets. In the two unattractive stimuli, however, additional spike bursts occurred in the middle or at the end of the syllables. The AN1-neuron marked the syllable onsets of the standard stimulus, whereas the perturbations evoked additional spikes within the syllables. The trial-averaged firing rates (Figure 3, color coded) of all recorded neurons indicate that neuronal response patterns vary for the four different song patterns. Also, neurons that are of the same morphological type (AN1, AN3, AN4, AN12) show variations in their response patterns across individual animals.

We use a Bayesian approach to classify the acoustic stimulus based on the neural activity (see Materials and Methods). To this end we counted the number of spikes in each single trial and for each of the four stimuli during the complete stimulus duration of 756 ms, comprising 9 syllables and the respective pauses. Based on the spike count we decoded the stimulus identity according to the classification rules in Different Decoding Approaches. We measured the classification performance by the MCC.

**Figure 4:**
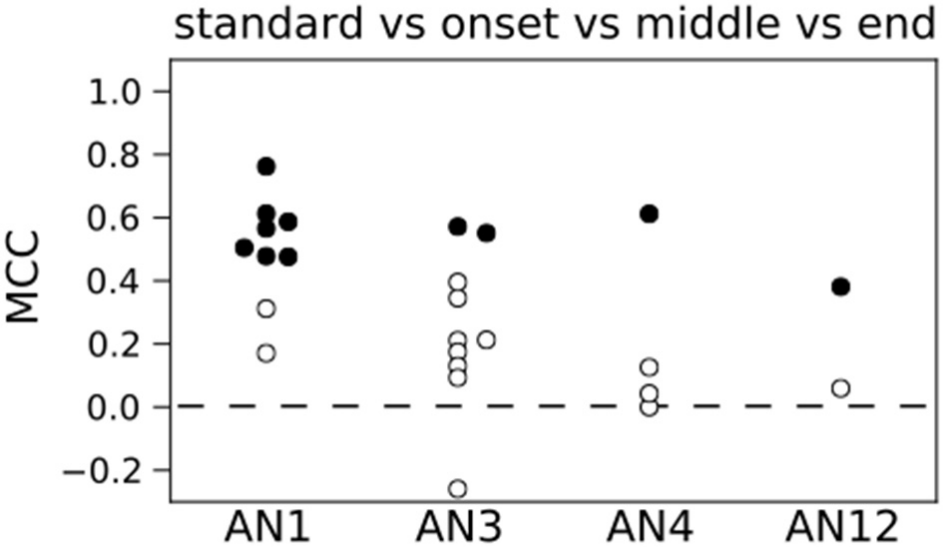
**Count based decoding of stimuli in single neurons**. A classification of the four stimuli is in 11 (filled circles) out of 25 neurons significantly better than a classification based on randomized counts. The distribution of MCC values of all 25 neurons differs significantly from the MCC distribution of the classifiers that are based on randomized counts (*p* < 0.05, one-sided Wilcoxon rank-sum test). Dashed line represents chance level based on randomized counts.

**Figure 5:**
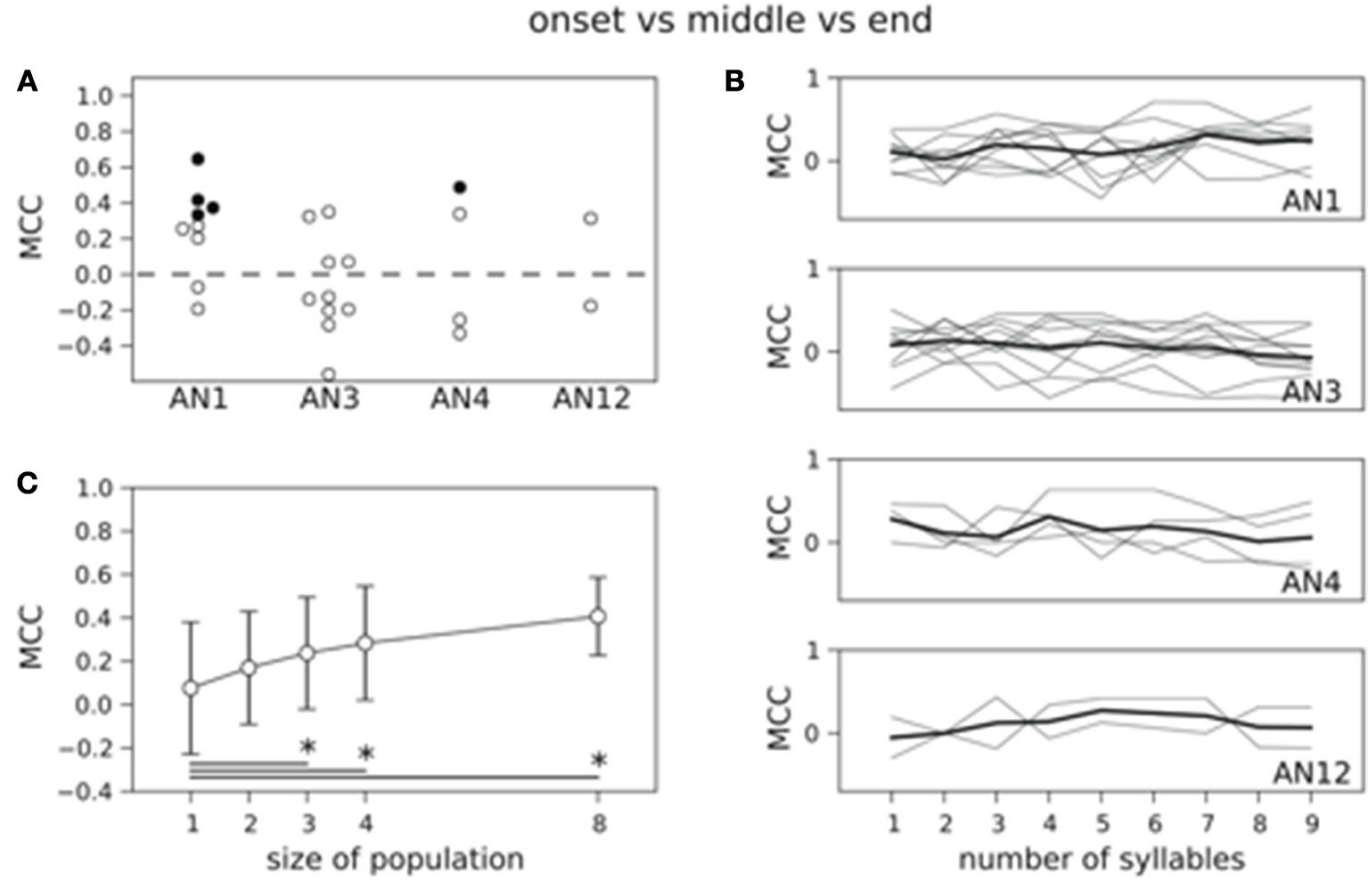
**Count based decoding of the three perturbed stimuli in single neurons and populations. (A)** Only in 5 neurons the three perturbed stimuli are decoded significantly better than a classification based on randomized counts. The distribution of MCC values of all 25 classifiers does not differ significantly from the MCC distribution of the classifiers that are based on randomized counts (*p* = 0.23, one-sided Wilcoxon rank-sum test). Dashed line represents chance level. **(B)** Averaged time course of the MCC is not increasing with stimulus duration (thick black line). **(C)** Decoding performance increases with population size. Classification is based on the spike count measured over all nine stimulus periods. MCCs are averaged across neurons and vertical error bars depict standard deviation. The mean performance increases significantly from single neurons to populations of size three, four, and eight (*p < 0.05, one-sided Wilcoxon rank-sum test).

Figure 4 shows the results for decoding the four stimuli from single neuron activity. The MCC was higher than chance level for all but two neurons (see Figure 4) and 11 out of 25 decoded the stimuli significantly better than on basis of randomized counts (black dots in Figure 4, *p* < 0.05). Averaging across all 25 neurons yielded a mean MCC of 0.32. The decoding results were best for the standard song (not shown). As shown in Figure 1A the standard song had a higher syllable plateau than the perturbed songs which is a consequence of our constraint that all stimuli have the same effective intensity (see Materials and Methods). A closer look showed that the trial-averaged spike count elicited by the standard syllables differed from the spike counts evoked by the perturbed syllables. However, this is not consistent across neurons. For some neurons the spike count evoked by the standard stimulus is considerably larger than the spike count evoked by any of the perturbed stimuli, for other neurons this relation is reversed. This difference between the spike count triggered by the standard and the perturbed stimuli is reflected in a higher performance in decoding the standard stimulus against the class of perturbed stimuli (Figure S1: averaged MCC is 0.78; 22 neurons decode significantly better than by chance). To avoid a bias of the decoding performance due to the higher syllable plateau of the unperturbed standard stimulus, we restrict our analyses to the stimulus set of the three perturbed songs throughout the rest of the manuscript. This reduced stimulus set yielded only 5 neurons that allowed for a successful decoding of the three stimuli, and the average MCC dropped sharply to 0.08 (Figure 5A).

So far, the spike count was measured during the complete stimulus presentation which consists of nine periods (syllable plus pause). Next, we asked how good we can decode the stimuli based on the spike count extracted over shorter time windows. To this end, we investigated the MCC as a function of the number of periods starting at stimulus onset (Figure 5B). Interestingly, the MCC, averaged across neurons within one class, stayed constant over stimulus time (see thick lines in Figure 5B). For single neurons the MCC fluctuated without apparent increase or decrease (thin lines in Figure 5B).

The performance of the Bayesian classifier generally depends on the encoding rate signal and on the noise that is evident in the trial-by-trial variability of the spike train responses. High variability increases the uncertainty of the decoder model. We estimated the trial-by-trial spike count variability in our AN recordings using the Fano factor (see Materials and Methods). As shown in Figure S2 the variability remained constant with increasing the stimulus time in almost all neurons. This fits the result of the constant decoding performance independent of stimulus duration in Figure 5B.

As the grasshopper brain receives input from several ANs (up to 20 at each side Stumpner and Ronacher, 1991) we next decoded the three perturbed songs from neuronal populations (see Materials and Methods). We constructed neuronal populations up to size four with each neuron from a different type, representing a subpopulation of ANs in one hemisphere. Additionally, we decoded on a basis of populations of size eight, consisting of two different neurons of each available type reflecting the input from both ears. As to be expected the averaged decoding performance is increasing with population size up to an average MCC = 0.41 for 8 neurons if counts were extracted over the complete stimulus duration (Figure 5C). This improvement was significant between populations of size 3 or larger and single neurons (*p* < 0.05, one-sided Wilcoxon rank-sum test).

#### Decoding of the behavioral relevance

**Figure 6:**
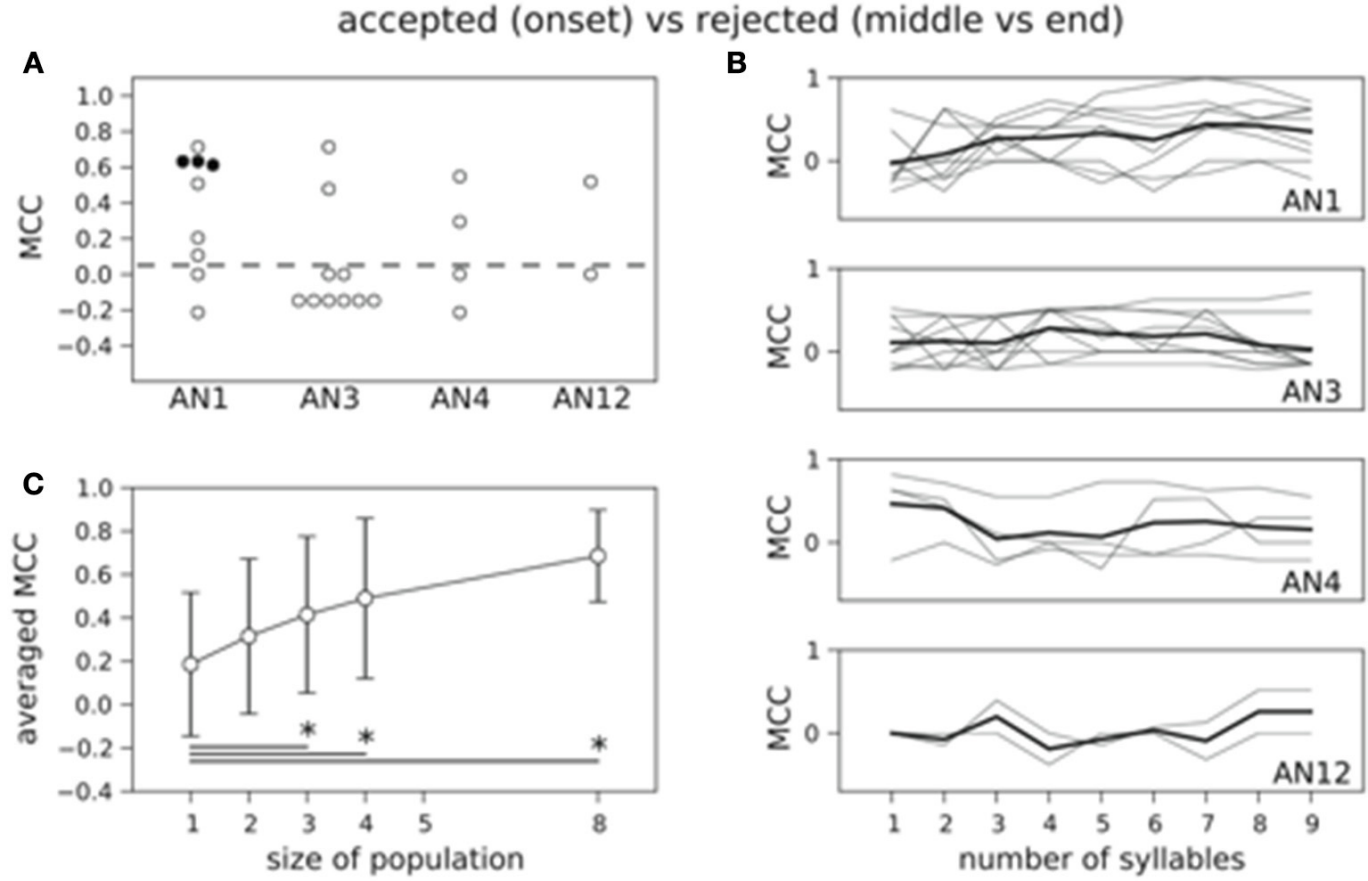
**Count based decoding of behaviorally relevant classes in single neurons and populations. (A)** Decoding the class of accepted versus the class of rejected stimuli is in only 3 neurons successful. The distribution of the 25 MCC values does not differ significantly from the MCC distribution of the classifiers that are based on randomized counts (*p* = 0.45, one-sided Wilcoxon rank-sum test). **(B)** Averaged time course of the MCC for each neuron class is not increasing with stimulus duration (thick black line). Gray lines indicate results for single neurons. **(C)** Decoding performance increases with population size. The increase differs significantly between single neurons and populations of size three and larger (*p < 0.05, one-sided Wilcoxon rank-sum test).

In our behavioral experiments stimuli fell into two behaviorally relevant classes: the standard song and the onset-perturbed song were attractive whereas songs with middle- and end-perturbed syllables were rejected (Figure 1B). Here we asked: is it possible to predict whether a song belongs to the accepted or rejected class based on the neuronal spike count? We again used a Bayesian decoder and evaluated the success of correct predictions in single trials with the MCC. We first considered the total spike count over all nine periods in single neurons. Only half of all MCC values were larger than zero and the number of neurons that decoded significantly better than by chance was reduced to 3 (Figure 6A). The MCC averaged across all 25 neurons was 0.19 and the distribution of the MCC did not differ significantly from the distribution of the performance values based on randomized counts (*p* = 0.45, one-sided Wilcoxon rank-sum test). Investigating the MCC as a function of the number of periods starting at stimulus onset again showed a constant representation across syllables (cf. Figure 6B).

If information was used from AN populations, the performance improved remarkably up to an average MCC of 0.69 (counts over all nine periods; Figure 6C) for populations of size eight. This increase differed significantly between single neurons and populations of size three or larger for counts measured over the complete stimulus duration (Figure 6C). Our results show that information about the behavioral relevance is encoded in the time-averaged AN population rate.

### Modeling the behavioral decision based on sensory evidence

Thus far we have shown that a population of ANs carries a significant amount of information about the behavioral relevance of the stimulus that allowed for a binary classification of the attractive vs. the unattractive stimulus class based on the neurons' spike count (Figure 6C). Here we introduce a simple model of decision making inspired by Gold and Shadlen (2007). In our model we interpret the population spike count of the ANs as sensory evidence about the behaviorally relevant cues that indicate an attractive calling song (see Materials and Methods). Our results in Figure 6B indicate that this information is encoded in a persistent and stable manner across syllables. We thus hypothesize that a decision circuit at a higher processing level makes use of this stable representation at the sensory level by accumulating evidence across successive syllables.

Formally, our model (c.f. Materials and Methods, Model of Decision Making) assumes that the AN population firing rate for each syllable provides an independent piece of evidence about the behaviorally relevant cues. For each single trial spike count pattern in a population of 8 neurons and for each syllable separately we computed the log LR for the presence of an attractive song over the presence of an unattractive song. In a second step we integrated the log LR across syllables. We then define the decision variable (DV) as the time integral over the log LR. Positive values of the DV indicate that the presence of an attractive stimulus is more likely than the presence of an unattractive stimulus and vice versa for negative values of the DV.

**Figure 7:**
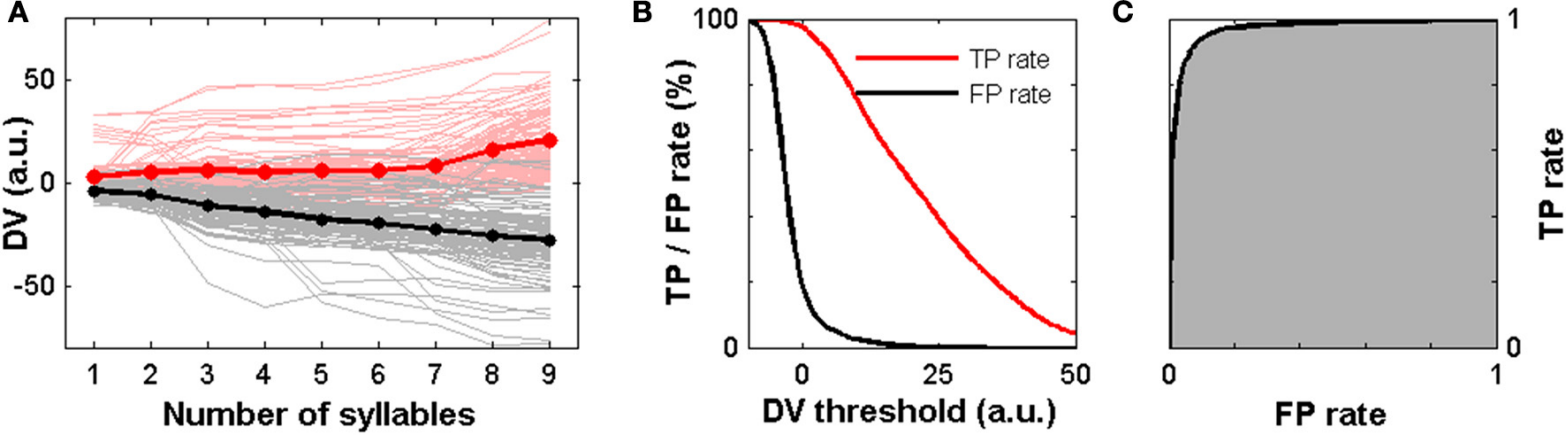
**A model of decision making based on the experimental spike trains. (A)** The DV as a function of the song syllables for 100 presentations of an attractive song (perturbed at the beginning of each syllable) are shown as light red lines. The average (red line) is computed across all possible combinations of 8 neurons and all trials. It signifies an overall increase over time. The single trial DV for 100 unattractive song presentations are shown as gray lines. The average (black line) shows a monotonic decrease over time. **(B)** TP rate (red) and FP rate (black) in dependence on the decision threshold computed across 9720 different combinations of 8 neurons and all single trial stimulus presentations. **(C)** The ROC (black line) relates TP rate and FP rate for a varying threshold. The area under the ROC (gray) amounts to 0.97.

Figure 7A shows the DV as a function of time based on the measured neuronal response patterns. In the case of attractive calling songs (red) the average DV is positive already during the first syllable and shows an overall increase over the 9 syllables. For trials in which an unattractive song was presented the average DV (black) steadily decreased across syllables. The individual single trial curves of the DV show a variable behavior (Figure 7A). In order to simulate the behavioral decision we introduced a decision threshold on the DV. In each single trial a response is simulated if the log LR value crosses this threshold during any of the syllables.

In the cases of an attractive (unattractive) trial we count a threshold crossing as TP or FP result, respectively. We then computed the TP and FP rates in dependence on the threshold value. The TP rate of the model relates to the female response rate for attractive song presentations in animal experiments, the FP rate relates to the female response rate to unattractive songs (Figure 1B). As shown in Figure 7B the FP rate drops sharply and much faster than the TP rate when increasing the decision threshold.

How does the model performance compare quantitatively to the behavioral experiments? The median female response rates were 83% for attractive stimuli and 6% for unattractive stimuli (Figure 1B). A variation of the decision boundary in our model corresponding to a variation of the TP rate in the range of 80–85% corresponds to FP rates in the range of 3–4% (Figure 7B). This indicates that the behavioral decisions based on the neural recordings from a population of 8 ANs and the simple decision model presented here are, on average, comparable to the average performance in the behavioral experiments with female grasshoppers.

The ROC in Figure 7C quantifies the model performance independent of the threshold. Integrating over the ROC (area under ROC) yielded a high value of 0.97 indicating that this decision model based on the neuronal population spike count performs very well in making correct detections of attractive calling songs and in avoiding false alarms in the case of unattractive calling songs.

## Discussion

### Population rate code at the output of the grasshopper thoracic pathway

We evaluated the information about stimulus and behavioral contingency using a simple measure of neuronal activity: the total spike count during stimulus presentation. For single neurons we obtained only poor decoding performances. The full time-resolved firing rate estimate over the stimulus duration carries much more stimulus information and naturally results in much higher decoding performances (Figure S3). However, in the realistic scenario of decoding the spike counts from a population of neurons the performance increased significantly as compared to the single neuron case. For the maximum population size of 8 ANs we obtained on average MCC = 0.69 for predicting the behavioral class (Figure 6C). We grouped maximally 8 neurons, two of each of the morphological types that had been recorded in our experiments. This represents a realistic subpopulation of ANs from an individual animal. We can expect that the decoding from an intact population of at least 20 morphologically distinct ANs per hemisphere in the grasshopper would reach considerably higher decoding performances, indicating that the relevant stimulus features are represented by a combinatorial rate code in the AN population. These results are particularly interesting in view of recent papers investigating different aspects of the grasshopper's auditory pathway. Clemens et al. (2011) provided evidence that between the local and ascending neurons, i.e., between the second and third processing stage, the coding principle changes from a summed population code to a labeled-line population code where the population's information is maximal if a decoder takes into account neuronal identity. At the level of the AN population, the temporal sparseness as well as the population sparseness increases (Clemens et al., 2012). At the same time, integrated spike rate information gains in significance compared to spike timing information (Clemens et al., 2011, 2012; see also Wohlgemuth and Ronacher, 2007; Creutzig et al., 2009; Ronacher, 2014) which fits our results. In addition, the use of a spike count code would also explain why the remarkable imprecise spike timing found in ANs (Vogel et al., 2005) does not impair the precise evaluation of song features in the millisecond range as observed in behavioral tests (Von Helversen, 1979; Ronacher and Stumpner, 1988; Ronacher and Stange, 2013; Ronacher, 2014).

### Persistent and reliable sensory evidence at the level of ascending neurons

We found that the across syllables information is encoded persistently and reliably in the AN population rate and we hypothesize that the role the grasshopper's auditory system is to provide stable sensory evidence that can be evaluated in the brain. The performance of the Bayesian classifier depends on both, the encoding rate signal and the noise. We found that the Fano factor of ANs, which estimates the noise as trial-by-trial variability of the spike number (Nawrot, 2010), is constant across time, indicating a constant level of noise in the peripheral auditory system (Figure S2). The absolute values of the Fano factor match previous results showing that variability of spike trains increases from receptor neurons to the ANs (Ronacher et al., 2004; Vogel et al., 2005; Vogel and Ronacher, 2007; Neuhofer et al., 2011), which on average showed a reduced performance in stimulus classification compared to LNs (Wohlgemuth and Ronacher, 2007). Using song models that were progressively degraded, Neuhofer et al. (2011) could estimate the respective contributions of external signal degradation and the trial-to-trial variability of spike trains caused by intrinsic neuronal noise. Intrinsic neuronal noise had a very strong impact on the spike train variability, in particular in ANs, thus likely affecting the representation of acoustic signals along the auditory pathway, and thus also the discrimination and recognition of grasshopper songs (Ronacher, 2014).

### Integrating sensory evidence for behavioral decisions—a hypothetical brain algorithm in the grasshopper

At the level of ANs that provide the sole auditory input to the grasshopper's brain we found a steady representation of information about the stimulus and its behavioral relevance in the population spike count. We devised a simple decision making model that integrates evidence over time generating a decision variable, which eventually may reach a decision threshold to elicit a behavioral response. Such models have previously been formulated for alternative choices in sensory decision tasks (e.g., Gold and Shadlen, 2007; Beck et al., 2008; Drugowitsch and Pouget, 2012). The model integrates the estimated Bayesian likelihood across successive syllables and, by crossing a decision threshold allows to form behavioral decisions. In the grasshopper, recognition, and evaluation of a conspecific calling song simplifies to the female's decision between showing or not showing her response behavior depending on whether and when the evidence reaches a threshold. In a neuroethological context as well as in controlled behavioral experiments animals can modulate their behavioral response level (Von Helversen and von Helversen, 1994, 1997; Wirmer et al., 2010). In our model this could be realized by a modulation of response threshold, e.g., through neuromodulators in the relevant brain circuit (Heinrich et al., 2001; Wirmer et al., 2010).

Our model presented here is based on neural recordings in the auditory pathway and thus extends on approaches that model female response behavior based on the auditory stimuli alone. Clemens and Ronacher (2013) devised an abstract linear-nonlinear cascade model: In a first step the model continuously extracts characteristic stimulus features from the sound stimulus by use of linear filters. In the second step the model transforms each filter output with a non-linear function. The resulting signals are then integrated across features and over the whole stimulus period, neglecting the exact temporal position of specific song features. Their model was able to predict behavioral responses with high reliability (*r*^2^ = 0.87) with a set of only two distinct song features. This serial structure of (i) extraction of sensory evidence, and (ii) subsequent temporal integration over this evidence is paralleled in our model and the model proposed by Clemens and Ronacher (2013).

If we assume a time-integrating algorithm in the grasshopper's brain, what could be the underlying neuronal mechanism? The relevant time span is indicated by the duration of the reported response times in the range of typically several hundreds of milliseconds. One cellular mechanism that could serve this task is short-term synaptic plasticity. Fascilitation and depression at synapses are governed by processes with typical time constants in the right order of magnitude and they have repeatedly been suggested to be involved in decision making processes (Mongillo et al., 2008; Martínez-García et al., 2011) including a suggested algorithm for auditory pattern recognition in the cricket's central brain (Rost et al., 2013).

In summary, our results support the hypothesis of a population rate code in ANs that project the acoustic information to the central brain (see Clemens et al., 2011, 2012). The information about the behavioral relevance of a stimulus is well represented in the population rate and this information is constantly present throughout the stimulus presentation. The good performance of our decision model suggests a computational process located within the grasshopper brain that infers the behaviorally relevant information and integrates this evidence over time to reach a behavioral decision based on accumulated evidence.

### Conflict of interest statement

The authors declare that the research was conducted in the absence of any commercial or financial relationships that could be construed as a potential conflict of interest.
