# Supplementary material for: Neural representation of calling songs and their behavioral relevance in the grasshopper auditory system
Source: Front Syst Neurosci. 2014 Dec 19;8:183. doi: 10.3389/fnsys.2014.00183 (PMC4271601; doi:10.3389/fnsys.2014.00183)
Supplement: Supplementary file 1 [file DataSheet1.DOCX]

***Supplementary Material***

**Neural representation of calling songs and their behavioral relevance in the grasshopper auditory system.**

**Gundula Meckenhäuser^1 +^,Stefanie Krämer^2 +^,Farzad Farkhooi^1^,Bernhard Ronacher^2^, Martin P Nawrot^1^***

^1^ Neuroinformatics & Theoretical Neuroscience, Institute of Biology, Department of Biology, Chemistry and Pharmacy, Freie Universität Berlin, Germany

^2^ Behavioural Physiology Group, Department of Biology, Humboldt-Universität zu Berlin, Germany

^+^ equal contribution

*** Correspondence:** Martin P Nawrot, Neuroinformatics & Theoretical Neuroscience, Institute of Biology, Department of Biology, Chemistry and Pharmacy, Freie Universität Berlin, Königin Luise Straße 1-3, Berlin, 14195, Germany.

martin.nawrot@fu-berlin.de

1. **Count-based decoding of standard versus perturbed stimuli.**

**
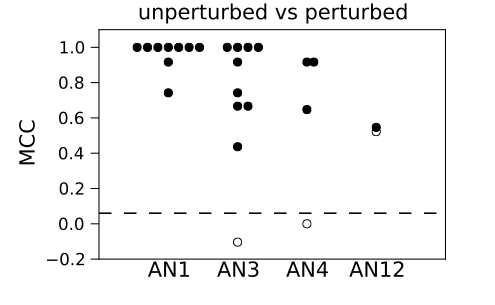
**

**Supplementary Figure S1. Classification of unperturbed vs. perturbed stimuli from the single neuron spike count.** A classification of the standard against the perturbed stimuli is in 22 (filled circles) out of 25 neurons significantly better than a classification based on randomized counts. The distribution of MCC values of all 25 neurons differs significantly from the MCC distribution of the classifiers that are based on randomized counts (p<0.05, one-sided Wilcoxon rank-sum test). Dashed line represents chance level based on randomized counts.

1. **Trial-by-trial variability of AN responses**


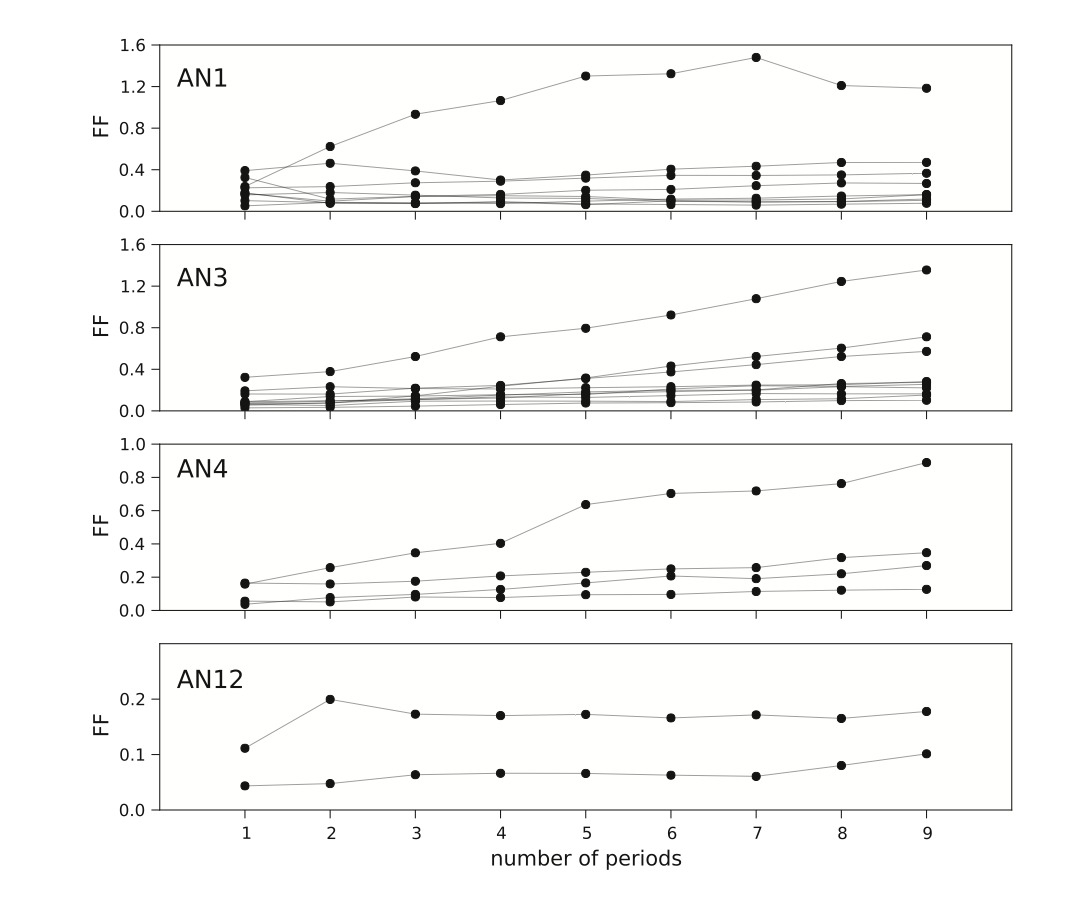


## Supplementary Figure S2. Spike count variability in single neurons. FF estimated separately for all neurons. Each panel corresponds to the respective AN type as indicated. Each line corresponds to a single neuron. The FF was computed for each of the four stimuli separately from the spike count across all trials in an interval that starts at stimulus onset and comprises the respective number of song periods. Each data point represents the average FF across all four stimuli.

We measured the trial-by-trial spike count variability in single ANs by the Fano factor (FF), which computes the ratio of the spike count variance and the mean spike count across all single trial responses to a given stimulus (Nawrot 2010). The results shown in Supplementary Figure 3 indicate that the FF stays constant in most neurons when increasing the estimation interval from 1 to 9 periods. The absolute numbers vary in the range of 0.05 to 0.5 except for a few outlier neurons that show high variability. This quantitatively matches previous estimates in independent AN recordings (Vogel et al. 2005; Vogel and Ronacher 2007; Neuhofer et al. 2011). These absolute values of the FF correspond to a spiking process that is clearly more regular and less variable than a Poisson process in general agreement with a wide range of spiking neurons that have been measured under stationary stimulus conditions (e.g. cortical neurons, Nawrot et al., 2008). The fact that ANs in the grasshopper show spike frequency adaptation (Hildebrandt et al., 2009) implies cellular mechanisms of self-inhibition that result in a comparably low spike count variability (Farkhooi et al., 2011; Farkhooi et al., 2013).

The time constancy of the FF is in agreement with the assumption of a stationary point process, i.e. with a fixed stochasticity of the neural spiking process, which represents a neuron’s intrinsic noise source, and with a stationary intensity or ‘drive’ of this process. This result of a time-constant FF thus supports the hypothesis of a persistent sensory representation of stimulus features (evidence) across syllables and it indicates a constant level of noise in the peripheral auditory system.

1. **Decoding time-resolved firing rates**

In the main body of the manuscript we computed the Bayesian probabilities based on the number of spikes (proportional to the averaged firing rate) as counted in a fixed time window that is large compared to the temporal variations in the song structure due to syllable perturbation.

Here we show the results for a Bayesian decoding from time-resolved firing rates in single neurons. Firing rates were estimated with millisecond resolution by convolving a spike train with a Gaussian kernel with kernel widths б. This yielded rate vectors $r_{0},\ldots,r_{756}$ from $t=0,\ldots,756$ms (see 2.5.1.). Fast changes in the spike rate are better represented if we estimate with small kernel widths (see (Figure 3). Using the single trial rate vectors we decoded the stimuli according to

$\hat{s}= {argmax}_{s\in S}\left\{ \prod_{t=0}^{t=756} P\left( r_{t} | s \right)P(s) \right\}$.

Here, $P\left( r_{t} | s \right)$ was estimated as explained in 2.5.2.


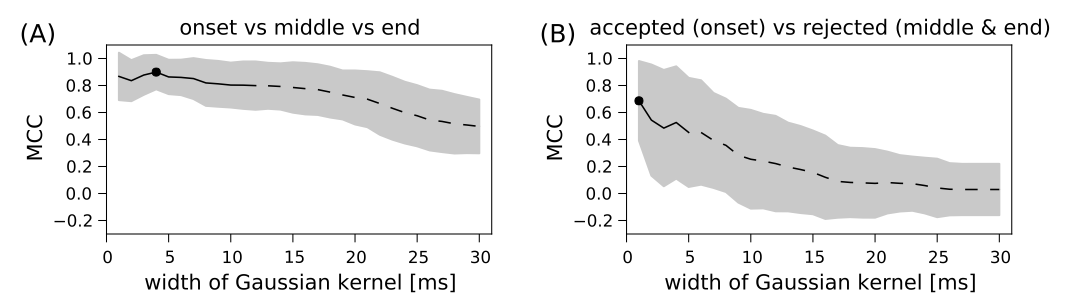


**Supplementary Figure S3. Time-resolved firing rate based decoding in single neurons.** Averaged performance is shown as a function of the width of the Gaussian kernel that was used for estimation of the firing rates. Grey area shows standard deviations. Classifiers are best performing with firing rates determined with high temporal resolutions (Gaussian kernel of widths б=1,…,12ms (A) and б=1,…,5ms (B) and within these ranges (solid lines) they do not differ significantly from the best ones (black circles; p=0.05, two-sided Wilcoxon rank-sum test).

Supplementary Figure S3 shows the performance of single-trial classification averaged across neurons as a function of б. Classifying the three perturbed stimuli yielded good performances for rate estimates with kernels widths σ in the range of 1,…, 12ms (continuous line in Figure S3A). The average MCC reaches a maximum of 0.89 at σ = 4ms (see filled circle in Figure S3A). This result underlines the differences in the temporal structure of the spike patterns evoked by the three stimuli (see Figure 3) and the optimal σ of 4 ms corresponds to the length of the perturbations, which is 2 σ=8ms. Decoding behaviorally relevant classes (accepted versus rejected songs) performed best (MCC=0.68) with σ = 1ms, and σ = 1,…, 5ms yielded classifiers with similar performance (not significantly different; SF 3B). However, the range of MCC covered by individual neurons (grey area in Figure S3B) was much larger than in Figure S3A, indicating that some neurons performed well in this task whereas others performed badly.

As to be expected, the classification performance is overall much improved when considering time-resolved rate vectors as compared to the overall spike count. The rate vector with millisecond resolution holds considerably more information than the time-averaged rate. It captures all modulations of the firing rate that closely follow the stimulus dynamics, in particular it captures the differences in the perturbation structure (Figure 1).

However, this result needs to be evaluated with caution. When training and testing our Bayes classifier we use the stimulation onset as a temporal trigger for the alignment of the response spike train. Random scattering of the stimulus onset during training and testing would strongly impair the classification performance. The stimulus onset trigger is experimentally defined with millisecond precision. However, the animal does not have any independent datum about the stimulus onset. It evaluates the song in an online fashion and would need to make an accurate estimate of its onset. Moreover, it would be difficult to envision a neuronal algorithm in the grasshopper brain that evaluates the time-resolved firing rate vector in a manner similar to a machine learning algorithm that is presented with a snapshot image of the time-dependent rate function.

1. **References**

Farkhooi F, Müller E, Nawrot MP (2011) Adaptation reduces variability of the neuronal population code. Physical Review E 83: 050905.

Farkhooi F, Froese A, Müller E, Menzel R, Nawrot MP (2013) Cellular Adaptation Facilitates Sparse and Reliable Coding in Sensory Pathways. PLoS Computational Biology 9: e1003251. doi:10.1371/journal.pcbi.1003251

Hildebrandt, K. J., Benda, J., & Hennig, R. M. (2009). The origin of adaptation in the auditory pathway of locusts is specific to cell type and function. The Journal of Neuroscience, 29(8), 2626-2636.

Nawrot, M. P., Boucsein, C., Rodriguez Molina, V., Riehle, A., Aertsen, A., and Rotter, S. (2008). Measurement of variability dynamics in cortical spike trains. *Journal of Neuroscience Methods*, 169(2), 374-390.

Nawrot, M. P. (2010). ''Analysis and interpretation of interval and count variability in neural spike trains'' in *Analysis of parallel spike trains* eds S. Grün and S. Rotter (Springer US), 37-58.

Neuhofer, D., Wohlgemuth, S., Stumpner, A., and Ronacher, B. (2008). Evolutionarily conserved coding properties of auditory neurons across grasshopper species. *Proc. R. Soc. B*, 275(1646), 1965–74. doi:10.1098/rspb.2008.0527.

Vogel, A., Hennig, R. M., and Ronacher, B. (2005). Increase of neuronal response variability at higher processing levels as revealed by simultaneous recordings. *J Neurophysiol*, 93, 3548–59. doi:10.1152/jn.01288.2004.

Vogel, A., and Ronacher, B. (2007). Neural correlations increase between consecutive processing levels in the auditory system of Locusts*. J Neurophysiol*, 97, 3376–3385. doi:10.1152/jn.00796.2006.
